# With a little help from DNA barcoding: investigating the diversity of Gastropoda from the Portuguese coast

**DOI:** 10.1038/srep20226

**Published:** 2016-02-15

**Authors:** Luísa M. S. Borges, Claudia Hollatz, Jorge Lobo, Ana M. Cunha, Ana P. Vilela, Gonçalo Calado, Rita Coelho, Ana C. Costa, Maria S. G. Ferreira, Maria H. Costa, Filipe O. Costa

**Affiliations:** 1 University of Minho, Centre of Molecular and Environmental Biology (CBMA), Campus de Gualtar, 4710-057, Braga, Portugal; 2 Helmholtz-Zentrum Geesthacht, Centre for Materials and Coastal Research, Geesthacht, 21502, Germany; 3 L3 Scientific Solutions, Runder Berg 7a, 2502 Geesthacht, Germany; 4 Lisbon New University, MARE-Marine and Environmental Sciences Centre, Department of Environmental Sciences and Engineering, Faculty of Science and Technology, 2829-516 Monte de Caparica, Portugal; 5 University Lusófona, Department of Life Science, School of Psychology and Life Sciences, Campo Grande 376, 1749-024 Lisbon, Portugal; 6 Portuguese Institute of Malacology (IPM), Zoomarine, E.N. 125 km 65, Guia, 8201-864 Albufeira, Portugal; 7 The H Dubai - Office Building, Office 1602, P.O. Box 128846, Sheikh Zayed Road, Dubai, U.A.E; 8 University of the Azores, InBIO – Associate Laboratory, CIBIO-Açores, Centre of Investigation of Biodiversity and Genetic Resources, Biology Department, 9501-801 Ponta Delgada, Azores, Portugal

## Abstract

The Gastropoda is one of the best studied classes of marine invertebrates. Yet, most species have been delimited based on morphology only. The application of DNA barcodes has shown to be greatly useful to help delimiting species. Therefore, sequences of the cytochrome *c* oxidase I gene from 108 specimens of 34 morpho-species were used to investigate the molecular diversity within the gastropods from the Portuguese coast. To the above dataset, we added available COI-5P sequences of taxonomically close species, in a total of 58 morpho-species examined. There was a good match between ours and sequences from independent studies, in public repositories. We found 32 concordant (91.4%) out of the 35 Barcode Index Numbers (BINs) generated from our sequences. The application of a ranking system to the barcodes yield over 70% with top taxonomic congruence, while 14.2% of the species barcodes had insufficient data. In the majority of the cases, there was a good concordance between morphological identification and DNA barcodes. Nonetheless, the discordance between morphological and molecular data is a reminder that even the comparatively well-known European marine gastropods can benefit from being probed using the DNA barcode approach. Discordant cases should be reviewed with more integrative studies.

The Gastropoda, the largest class of the Mollusca, is one of the most abundant and diverse groups of marine species^1^,^2^,^3^. The number of described marine gastropods was estimated to be between 32,000 and 40,000 species, although this percentage represents probably only between 23 and 32% of the total estimated number of marine gastropods^4^. This diversity, compounded with historical reasons, has made the Gastropoda one of the best-studied animal groups^2^. In the Renaissance, the beauty and the facility of maintaining and storing seashells made them a target for collectors^5^. In Europe, the numerous “shell cabinets” of the royals and rich families, considered a symbol of status, led to the proliferation in the 18^th^ and 19^th^ centuries, of scientific expeditions aiming to collect shells for trade by specialised companies such as *Shell* (http://www.shell.com). This “shell predilection” was also the basis for the establishment of dedicated journals^5^, providing great stimulus to the study of molluscs.

In spite of being a well-studied group, many aspects of the taxonomy, systematics, and evolutionary relationships among and within some gastropod clades are poorly resolved^3^,^6^. In addition, identification of specimens and delimitation of species in this class are still largely based on the morphological characters of the shell, operculum and radula^7^. The delimitation of species based solely on morphological characters led, in some cases, to an overestimation of species because some groups exhibit high phenotypic plasticity, e.g. *Littorina saxatilis* (Olivi, 1792)^8^. This ability of a genotype to change its phenotype according to local environmental conditions^9^, does not reflect, in some cases, differences with taxonomic value among evolutionary lineages^8^. In other cases, morphological stasis masks the existence of either distinct evolutionary lineages or cryptic species, and leads to underestimation of the number of species^10^. The inaccuracy of specimens’ identification has posed limitations to research in some areas, such as biodiversity and biogeography, which rely on accurate taxonomy. Therefore, higher precision on species diagnosis is of paramount importance^11^. Nevertheless, in the last decade there have been efforts to use integrative approaches to improve the taxonomy of some genera and species of the Gastropoda e.g.^6^,^7^,^12^,^13^ and to resolve the phylogenetic relationships among clades^2^,^3^,^14^.

DNA barcoding has been successfully used in several gastropod families, to overcome the hurdles posed by the identification of specimens and delimitation of species based only on morphological characters^7^,^15^,^16^. The effective molecular identification of specimens requires the creation of a comprehensive reference library of DNA barcodes matching each morpho-species to a diagnostic barcode sequence or array of sequences^17^,^18^. To this end, a globally accessible DNA barcode database (BOLD system)^19^, comprising reference libraries generated by several researchers/institutions, is available for scrutiny by the scientific community and to validate new sequence data produced.

There are no recent compilations on the number of gastropod species in the Portuguese coast, in spite of the fact that many gastropod species are exploited commercially. In some cases, even overexploited to the brink of extinction, as for example the endemic patellid species in the Azores^20^. The last compilation by Macedo and colleagues^21^ dates from 1999 and reports 1105 gastropod species, but G. Calado (Portuguese Institute of Malacology, IPM) estimates that the number may vary between 1000 and 1500 species. Furthermore, the north-south orientation of the Portuguese coast, with a cooler northern region affected by upwelling and a high volume of river run-off, compared to a much warmer southern region with a strong Mediterranean influence^22^, makes it a unique area of confluence of cold- and warm-water species^23^. Indeed, many cold- and warm-water species have their southern and northern boundaries, respectively, in Portugal, making it a very important area for biogeographic studies (e.g.^24^,^25^,^26^). Hence, the aims of this study were to investigate the molecular diversity of gastropods’ morpho-species that were collected on the Portuguese coast (intertidal and subtidal), initiating a DNA barcode library for this taxon and region. In this process, we investigated the congruence between morphology-based identifications and DNA barcodes, and between the data generated herein and published sequences from other studies. We also gained insights into systematic relationships among species and phylogeographic patterns in some species.

## Results

### Morphological identification of species

**Figure 1:**
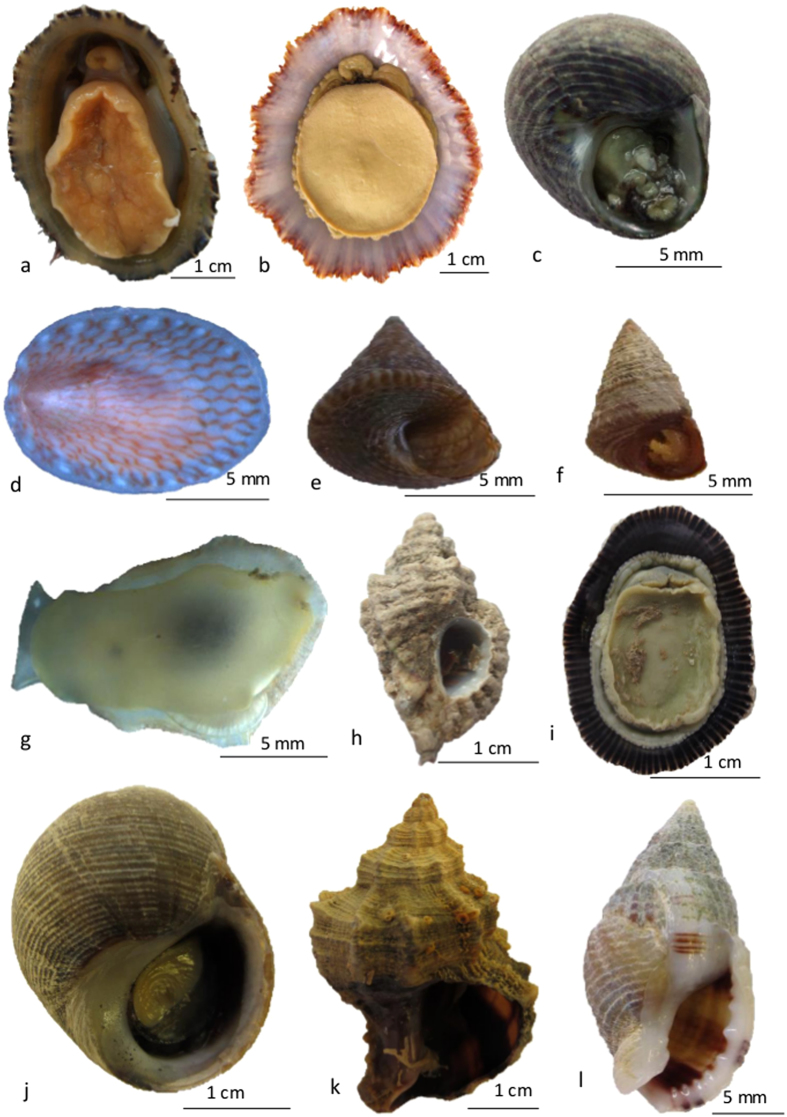
Selected images of gastropod specimens used in this study. (**a**) Patella ulyssiponensis; (**b**) Patella aspera; (**c**) Gibbula pennanti; (**d**) Tectura virginea; (**e**) Calliostoma virescens; (**f**) Jujubinus pseudogravinae; (**g**) Berthella plumula; (**h**) Ocenebra erinaceus; (**i**) Siphonaria pectinata; (**j**) Littorina littorea; (**k**) Hexaplex trunculus; (**l**) Nassarius reticulatus.

Morphological identifications to species-level were possible for 102 out of the 109 specimens in our dataset, comprising 34 morpho-species (some are illustrated in Fig. 1). These species were distributed across ten orders: Patellogastropoda, Vestigastropoda, Docoglossa, Neogastropoda, Archaeogastropoda, Sorbeoconcha, Littorinomorpha, Nudibranchia, Pulmonata, and Opisthobranchia, including 16 families, and 21 genera. Of all specimens, about 76% were collected from the continental coast of Portugal, while the remaining ones were collected from the Azores Islands. The number of specimens analysed per species varied from 2 to 10, but six species were represented by a single specimen. Only six genera were represented by more than one species, namely *Calliostoma*, *Gibbula*, *Littorina*, *Nassarius*, *Patella* and *Phorcus*.

### Species delimitation using COI-5P sequences

Of a total of 113 sequences in this project, 109 were barcode compliant (sensu BOLD^19^). Four sequences were not included in the dataset, three were reported either as the result of misidentification or contamination, and one did not meet BOLD compliance standards. In addition to the data obtained in the present study, we included sequences of 10 specimens previously published by Lobo and co-authors^27^, and 104 COI-5P sequences mined from GenBank. The final dataset comprised 223 COI-5P sequences, which were assigned to 58 morpho-species (supplementary Table S1; dataset container Gastropoda of Portugal, GOP, publicly available in the BOLD system).

**Figure 2:**
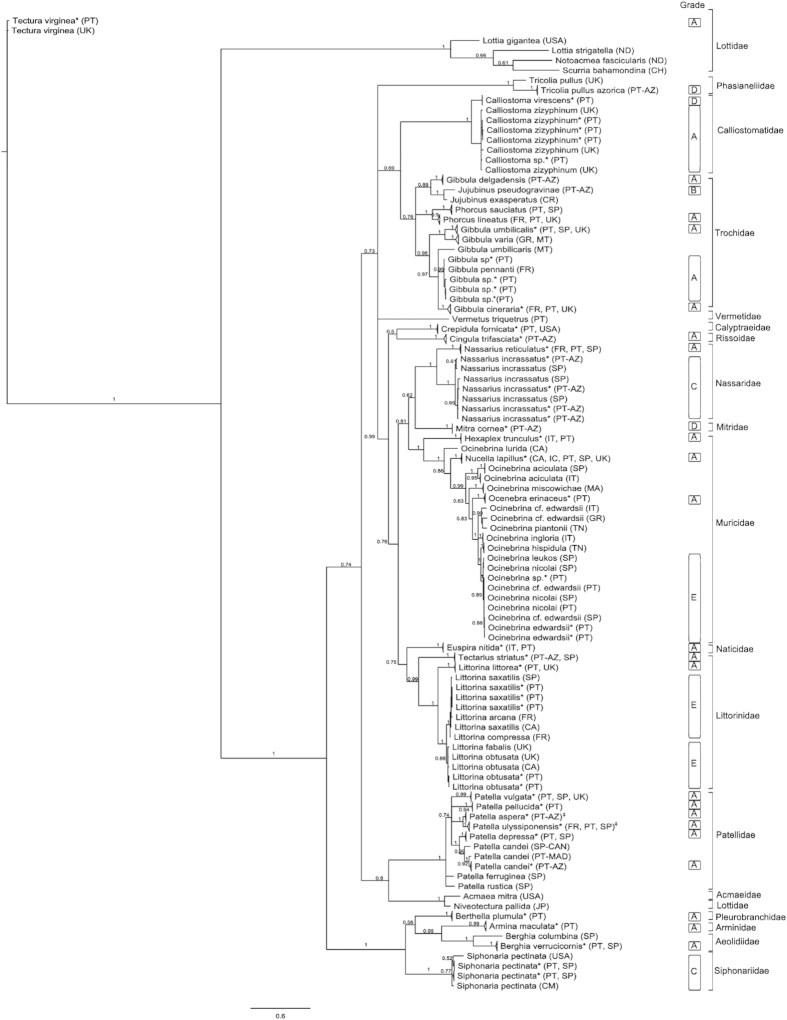
The Bayesian tree based on the COI-5P region. Numbers associated with nodes represent posterior probabilities from Bayesian MCMC searches conducted in MrBayes. Asterisk indicates sequences obtained in this study. ^‡^indicates branches with sequences obtained from GenBank with outdated species names (see supplementary Table S1 for the original names). CA- Canada; CM- Cameroon; CR- Croatia; FR- France; GR- Greece; IC- Iceland; IT- Italy; JP-Japan; MT- Malta; MA- Morocco; ND- No data; PT- Portugal; PT-AZ- Portugal, Azores; PT-MAD- Portugal, Madeira; SP- Spain; SP-CAN- Spain, Canary Islands; TN- Tunisia; UK- United Kingdom; USA- United States of America.

In 95% of the specimens, the COI-5P sequences were longer than 600 bp. The amino acid translation of the sequences showed no frameshift mutations, stop codons or unusually divergent amino acid profiles in the alignment. The similarity searches in GenBank and BOLD returned a significant identity match (99–100%) for 73% of the sequences in our dataset. Novel barcodes were produced for five species, *Calliostoma virescens* Coen, 1933, *Cingula trifasciata* (Adams, 1800), *Gibbula delgadensis* Nordsieck, 1982*, Jujubinus pseudogravinae* Nordsieck, 1973, *Mitra cornea* Lamarck, 1811, and one subspecies *Tricolia pullus azorica* (Dautzenberg, 1889). For those, the nearest match was found at genus or family level (83–88% similarity). Similarly, a low match was obtained for *Vermetus triquetrus* Bivona-Bernardi, 1832 (80%) from the study by Lobo and colleagues^27^. This sequence is the only one for this genus (*Vermetus*) in public databases. The identity of *Patella ulyssiponensis* Gmelin, 1791 and *Patella aspera* Röding, 1798 could not be clearly resolved by GenBank or BOLD searches. Both species displayed equally significant matches with one another. Some specimens that were identified morphologically only to genus, were later identified to species level based on their barcodes. For instance, *Gibbula* sp., *Calliostoma* sp., and *Nassarius* sp. grouped congruently in the clusters of *Gibbula pennanti* (Philippi, 1846)^12^, *Calliostoma zizyphinum* (Linnaeus, 1758), and *Nassarius incrassatus* (Strøm,1768), respectively (Fig. 2).

The Bayesian inference (BI) tree showed clearly defined and well supported clusters (Fig. 2). Some clusters, however, included more than one species. *Littorina saxatilis* clustered with other *L. saxatilis* from Spain and Canada, but also with *L. compressa* Jeffreys, 1865 and *L. arcana* Hannaford-Ellis, 1978 from France. When the sequences of *L. saxatilis* were submitted to a BLAST search in GenBank, sequences of *L. compressa* and *L. arcana* also showed as closes matches. Similarly, our specimens of *L. obtusata* (Linnaeus, 1758) clustered with specimens of *L. obtusata* collected in the UK and Canada, but also with *L. fabalis* (Turton, 1825) from the UK. The cluster including our specimens *of Ocinebrina edwardsii* (Payraudeau, 1826) was the one that contained the highest number of species. It includes species such *as O. hispidula* (Pallary, 1904), *O. leukos* Houart, 2000, and *O. nicolai* Monterosato, 1884, among others (Fig. 2).

COI-5P barcode distances for gastropods species analysed in this study are shown in Table 1. The BOLD’s distance summary was determined excluding cases of ambiguity, namely specimens that could not be morphologically identified to species level (e.g. *Gibbula* sp.; *Ocinebrina* cf. *edwardsii*), as well as species with invalid names retrieved from public repositories (e.g. *Osilinus lineatus*) because that would produce incorrect distance estimations. The intraspecific Kimura-2-Parameter (K2P) distances were inferior to 2% in the majority of the species with more than two analysed specimens. The mean intraspecific distances averaged 0.8% (range 0.0–6.38%), but two species displayed distances higher than 6%, namely *Patella candei* d’Orbigny, 1840 and *Siphonaria pectinata* (Linnaeus, 1758) due to the inclusion of one specimen from the Canary Islands, Spain and another from Florida, United States, respectively (Table 1). In addition, *Nassarius incrassatus* showed a maximum intraspecific distance of 3.5%, although the average intraspecific divergence in this species was lower, 1.9%. Sequence divergence for congeneric species ranged from 2.51 to 31.74% (mean 13.32%). The minimum distance was found between *Ocinebrina hispidula* and *Ocinebrina edwardsii* (within *O. edwardsii* complex), while the maximum distance occurred between *Lottia strigatella* and *Lottia gigantea*. The average confamilial distance was 17.84% (range 8.44–74.67%).

### Barcode Index Numbers (BINs) and ranking system for barcode records

The 109 barcode compliant sequences (34 morpho-species) were assigned to 35 BINs. The analysis of the “BIN report” produced by the BOLD system (as on 1^ST^ of July 2015) showed 23 concordant BINs, 11 discordant BINs and 1 singleton. The 11 discordant BINs were analysed in detail including: BLAST on GenBank to search for matching sequences for each of our sequences included in the BINs; consultation of authoritative databases, such as WoRMS or ITIS, to confirm the accepted names and synonyms for each species; checking the most up-to-date literature on the taxon/taxa included in the discordant BINs. After this, eight of the discordant BINs were considered concordant (AAA9623; AAA9624; AAB1828; AAO2227; AAO2228; AAZ9281; AAE1468; AAX5986), while three were considered discordant, still (AAO6343; AAA7469; AAG1552). This brings the number of concordant BINs to 32 (91.4%). For the PTP analyses, 35 putative species clusters were recovered for the 109 specimens in our dataset. The species were inferred based on the analyses of the most supported partition of the BI tree.

The application to our dataset of the ranking system proposed by Costa and co-authors^17^, and modified by Lobo and co-authors^28^, yielded twenty three species with grade A, one species with grade B, two species with grade C, five species with grade D, and 3 species with grade E (Fig. 2). The percentage of species scoring A and B was 70.5%.

## Discussion

Our COI-5P sequences were able to recover all morpho-species present in our dataset. In addition, the sequences of the same or related species (same genera) obtained either from GenBank or from the BOLD system, enabled us to compare and validate our results. As expected, K2P intraspecific average distance, including the sequences mined from public repositories was lower than 2% in all species. This shows that there is a good congruence between morphology-based identifications and DNA barcodes. However, in three morpho-species, *Patella candei*, *Siphonaria pectinata* and *Nassarius incrassatus*, maximum intraspecific K2P were higher (6.38%, 6.15% and 3.5%, respectively). The 2.2% maximum reference threshold for within-species boundaries, suggested by Ratnasingham & Hebert^29^ worked well for most of the taxa analysed, but its application cannot be generalised. To improve taxonomic resolution in the three latter morpho-species, it is important to increase the sampling effort, covering a wider geographic area and using integrative approaches that include sequence data from additional loci, among other sources of evidence. It is also important to look for new morphological characters that may help to distinguish cryptic species and, therefore, increase the taxonomic resolution in these species complexes.

In the case of the morpho-species *P. candei* and *S. pectinata* the occurrence of relevant genetic structure has already been reported in previous studies^30^,^31^. The taxonomic status of *P. candei* has long been considered uncertain, with four subspecies known to occur in the Macaronesian Islands. *Patella candei candei* has been reported upon in the Selvagens Islands (North Atlantic, between Madeira Archipelago and Canary Islands) and is considered to represent the ancestral form^30^. *P. c. ordinaria* is found in Madeira Archipelago, while *P. c. crenata* occurs in the Canary Islands^32^. The subspecies *P. c. gomesii* is considered endemic of the Azores^33^. The latter, is a hybrid with a high degree of introgression to *P. c. ordinaria*^30^. There is a high divergence between the subspecies *P. c. crenata* and *P. c. gomesii* (higher than 6%) and, therefore, the specimens appeared split in two clusters in the phylogenetic tree (Fig. 2). However, *P. c. ordinaria* (Madeira Archipealago) occurs in both clusters, sharing haplotypes both with *P. c. crenata* (Canary Islands) and with *P. c. gomesii* (Azores) (Fig. 2). Therefore *P. c. crenata* and *P. c. gomesii* probably represent extreme haplotypes of a complex genetic structure in *P. candei*^34^. *Siphonaria pectinata* has been considered an amphi-Atlantic species, but a genetic disjunction was shown to exist between western and eastern Atlantic populations, casting doubts on whether or not these two lineages constitute different species^31^. Our specimens of *S. pectinata* were divided in two BINs which corroborates the existence of two lineages. Similarly, *Nassarius incrassatus* has been reported upon as widely distributed in Europe, occurring from Iceland to Portugal, including the Mediterranean^35^ and the Azores (this study). Specimens of *N. incrassatus* were also divided in two BINs, although the average intra-specific K2P distance (1.9%) was below the 2.2% threshold used by the refined single linkage (RESL) algorithm^29^, which is applied in BIN assignments. However, in cases where sequences of the same morpho-species present clear variation, they are split over two or more BINs. This allows the separation of sets of sequences that might have been overlooked by a fixed value, and makes it possible to refine BIN attribution, when additional data are available^29^.

To further analyse the reliability of our sequences, we analysed the BIN report produced by the BOLD system. After reviewing the cases of BIN discordance, our library comprises 91.4% concordant BINs, showing a very good reliability of our results. In addition the 35 species suggested by the PTP analysis also show a good congruence with our BINs. The only exception was the case of the specimens of *Nassarius incrassatus*, which were divided in two BINs in the BOLD system but were considered a single species in the PTP analysis. The algorithm used to produce the BINs is probably more stringent than the one used to delimit the species in the PTP analysis. Thus, further studies should be conducted in this morpho-species to clarify the relevance of this high genetic distance among sympatric populations.

The incongruences found in the BIN report were due to three main reasons: 1) in some cases species names have not yet been updated in public repositories (e.g. GenBank; ITIS); 2) unstable taxonomy and species complexes in some morpho-species; 3) distinct species that were considered in some studies as a single species. Below we review the discordant BINs associated with our DNA barcode sequences, in light of the most up-to-date knowledge on each taxon analysed. Two BINs were discordant at genus level. *Phorcus lineatus* (da Costa, 1778), which included specimens of *Osilinus lineatus* (29), and Euspira, which included 1 specimen of *Lunatia*. However the valid genera are now *Phorcus* and *Euspira*, respectively (see WoRMS). Therefore, these BINs are now considered concordant. The BIN of *Ocenebra erinaceus* (Linnaeus, 1758) was considered discordant at species level because it includes specimens identified as *Ocenebra erinacea*. Similarly to the case above, the accepted species name is *Ocenebra erinaceus* both in the International Code of Zoological Nomenclature (ICZN) and in WoRMS, but *O. erinacea* is still considered the valid name in ITIS and some sequences mined from GenBank are also attributed to *O. erinacea*. The inconsistency between databases creates taxonomic problems and should be revised. Here we followed WoRMS and considered the BIN concordant.

The BIN including sequences of specimens identified morphologically as *Ocinebrina edwardsii*, was discordant at species level. In the northeastern Atlantic and Mediterranean *Ocinebrina edwardsii* is considered a species complex^36^ and is one of the most morphologically variable European muricids. The taxonomic status of the putative species included in this complex have been extensively debated (e.g.^37^). For this reason, *O. edwardsii* complex was recently the subject of a study using molecular markers (mitochondrial and nuclear) to attempt species delimitation^36^. In addition, in this study we included several species of this genus from other studies, available in GenBank^16^,^36^,^38^ (supplementary Table S1, Fig. 2). However, the BI tree shows a mix of morpho-species within neighbouring tree’s clusters that we have been unable to disentangle, even with the addition of extra specimens obtained from GenBank (Fig. 2). Hence definite conclusions cannot be drawn, and further studies are required to address this challenging species complex. Therefore, this BIN was considered discordant. The BIN containing our specimens of *Littorina saxatilis*, was also considered discordant at species level because of the inclusion of a specimen of *L. compressa* and another of *L. arcana*. The inclusion of the two latter species in the BIN can be explained by the non-monophyly of *L. saxatilis* revealed by several mtDNA markers (NADH1, tRNApro, NADH6 and partial cytochrome b)^39^ and COI-5P (this study). This BIN was considered discordant.

Also discordant was the BIN containing *Littorina obtusata*, which included also *L. fabalis*, one specimen identified only to genus level *Littorina* sp. and *L. saxatilis*. The presence of *L. saxatilis* in this BIN is probably the result of a misidentification or mis-labelling of the specimen because, as referred above, a large number of *L. saxatillis* occur in a separate BIN, and there is no ambiguity in the morphological distinction of the two species (*L. obtusata* and *L. saxatilis*). The specimen identified as *Littorina* sp. belongs to our dataset and was identified based on its DNA barcode as *L. obtusata*. The occurrence of *L. fabalis* in the BIN of *L. obtusata* was postulated to be the result of the invasion of a lineage of *Littorina* from the Pacific into the Atlantic, after the opening of the Bering Strait between 3.5–4 Myr^40^. These species occur sympatrically throughout their distribution range, but are well separated based on morphology, ecology and some genetic markers (allozymes and microsatellites)^41^. The lack of mitochondrial divergence between the two species^41^ is probably the result of a recent split^42^. In addition, results obtained by Kemppainen and co-authors^41^ also suggest that introgressive hybridization has occurred. Hence, in the case of *L. obtusata* and *L. fabalis* DNA barcodes cannot be used to delimit them. This BIN was also considered discordant.

Two BINs were considered discordant because of the inclusion of two specimens identified morphologically only to genus level, *Nassarius* sp. and *Siphonaria* sp., respectively. In both cases the two specimens can now be identified based on their barcode as *Nassarius incrassatus* and *Siphonaria pectinata*. The BIN including 4 sequences of *Gibbula* sp. (our study) matched 99.54% with a published sequence of a specimen of *G. pennanti*^12^. In addition, four unpublished sequences from *G. pennanti* are also present in the BIN. Hence, we can now identify our sequences of *Gibbula* sp. based on their barcodes as *G. pennanti*. The three BINs were considered concordant.

The most conflicting BINs were represented by *Patella aspera* and *Patella ulyssiponensis*, with two different BINs comprising both species. The occurrence of *P. ulyssiponensis* and *P. aspera* in both BINs stems from the fact that these have been considered in some studies as a single species, a problem that was detected in the present and previous studies (e.g.^43^). As a result, we found in GenBank sequences of specimens collected in continental Europe (Spain) identified as *P. aspera* and also specimens collected in the Macaronesian Islands (Azores) identified as *P. ulyssiponensis* (supplementary Table S1). However, those sequences clustered unambiguously with *P. ulyssiponensis* from mainland Portugal and *P. aspera* from the Azores, respectively (Fig. 2). Indeed, a morphological analysis and a study with allozymes showed that populations from Macaronesian and European continental populations were two distinct species^43^,^44^. It was proposed, therefore, to apply the specific status of *P. ulyssiponensis* for European continental populations (type locality, Lisbon, Portugal) and *P. aspera* for Macaronesian populations (type locality, Canary Islands, Spain)^43^. Moreover, a phylogenetic analyses carried out by Sá-Pinto and co-authors^14^, using mitochondrial genes, revealed two well supported clades within *P. ulyssiponensis (s.l.)*, one grouping all continental specimens and the other all specimens collected in the Macaronesian Islands. Later^45^, demonstrated that the absence of gene flow between the two populations led to a genetic disjunction. The genetic divergence between *P. ulyssiponensis* and *P. aspera* in the present study (4%) further supports that these are indeed two species, confirming the results of the studies mentioned above. The low K2P distance found between the two species is probably the result of a recent split, around 8.3–3.9 Million years (Myr)^14^,^45^. Taking into account our results and the previous evidence that *P. ulyssiponensis* and *P. aspera* are two different species, herein we used the nomenclature proposed by Weber and Hawkins^43^, and emphasize the importance of using the correct names to refer to them as proposed by various authors^43^,^44^,^46^. This is particularly important for regulation purposes in order to conserve the overexploited *P. aspera* in the Macaronesian Islands^20^.

The grades attributed to our species’ barcodes, according to the ranking system proposed by Costa and co-authors^17^, showed a high taxonomic reliability of our dataset with 22 morpho-species scoring A and 1 B. This shows a high general concordance between our sequences and sequences obtained for the same morpho-species in independent studies. *Nassarius incrassatus* and *Siphonaria pectinata* scored grade C showing the need for more integrative studies to determine whether these morpho-species are composed by more than one lineage (see above). Our barcode records that were attributed grade D are a reminder that the DNA barcode library initiated for this taxon in the present study is still in an initial phase, and much work is needed to bring it to completion. Three of our morpho-species scored grade E, namely *Littorina saxatilis*, *Littorina obtusata* and *Ocinebrina edwardsii*. There are all well-known species complexes that have been the subject of morphological, anatomical and molecular studies (see above), but further work is need, particularly in the latter species, to understand which of the species included in this complex are putative species and which should be considered synonyms.

The works by Smith *et al.* and Zapata *et al.*^2^,^3^ strongly support the monophyly of the Gastropoda, based on molecular analyses. Yet, the phylogenetic relationships among and within gastropod clades have remained instable^3^,^47^. COI-5P sequences have generally been used to infer relationships among groups with a more recent ancestry. However, studies in several molluscan genera showed that these sequences have the ability to recover somewhat deeper phylogenies^48^. In the present study we observed that the family Lottiidae is non-monoplyetic, which is in accordance with the results obtained by Remigio & Hebert^48^. In this family we detected a 3 bp (TTG) insert at nucleotide positions 94–96 and a 3 bp deletion in position 358–360 of the barcode region delimited by Folmer primers^49^, in the sequences of *Tectura virginea* (Müller, 1776) analysed in this study. Similarly, 3 bp inserts were observed in COI-5P sequences of the same and other species of Lottiidae^48^. In our study the insertions were *Tectura virginea* (TTG), *Lottia gigantea* Gray, 1834 (GGG), *Lottia strigatella* (Carpenter, 1864) (GGA), and *Lottia fascicularis* (Menke, 1851) (GGG). In neither case these length variants appear to represent pseudogenes as no termination codons were observed. The codon TTG produces the amino acid leucine in *T. virginea*, while the codons GGG, GGA and GGC produced glycine in *L. strigatella, L. fascicularis*, and *Scurria bahamondina*, respectively. Insertions and deletions (indels) can provide important information on phylogenetic relationships among taxa because they are less subject to homoplasy than nucleotide substitution^48^. Indeed, gastropods seems to have a much higher incidence of length variants than other invertebrate taxa^50^. However, further studies are needed to determine the impacts of these changes.

The barcodes produced herein for *Jujubinus pseudogravinae* and *Gibbula delgadensis* are the first for both species endemic of the Azores, and present in all Islands^51^. The Bayesian tree suggests that *G. delgadensis* is more closely related to *J. pseudogravinae* and *J. exasperatus* (a sequence obtained from GenBank) than to the other *Gibbula* species investigated in this study (Fig. 2). To confirm this pattern, and ascertain the systematic position of *G. delgadensis* in the genus *Gibbula* further investigation should be carried out, including the use of additional molecular markers and detailed morphologic examination. *Tricolia pullus azorica* is a subspecies endemic of the Azores^52^. A search for matching sequences in BOLD (as on 28^th^ of June 2015) returned a 94.9% match with specimens of *Tricolia pullus azorica*, collected in Galicia, Spain (3 private unpublished sequences). However, these specimens are well separated in two BINs, and display average COI-5P distances equivalent to what has been reported in this study for well-established species (e.g. *Littorina saxatlis* vs. *L. obtusata*). Therefore, it is improbable that the sequences from Galicia are of *Tricolia pullus azorica*. These two BINs are in turn more distant from its conspecific *Tricolia pullus* (Linnaeus, 1758), collected from Plymouth, UK^53^. The latter showed an even smaller maximum identity (84.6%) with *T. p. azorica* specimens from the Azores. Further research is needed to determine whether these subspecies should be elevated to the category of species.

## Conclusions

The barcode library presented herein intends to contribute to the great need of these libraries after the “genomics revolution”^18^. The use of technologies such as massive parallel sequencing of bulk samples (total DNA) are becoming increasingly common, but are often hindered by a shortage of comparable data from common loci^54^. The compilation of DNA barcode libraries is, however, time and resource consuming, as we experienced in the present study. One of the reasons is that in many cases the morphologic identification of species is difficult. In addition, there is a scarcity of taxonomists^55^, and a lack of detailed or updated taxonomic keys for several taxa, a basic requirement for taxonomists^56^. Nonetheless, the effort to produce such libraries is of great help for traditional taxonomy^18^. DNA barcodes can help, for instance, with problems of synonymy, which are particularly acute in the Gastropoda, the marine group where the number of species and the names for each species are more at odds, with 4 or 5 names for every species^1^. In addition, DNA barcodes can probe the comparatively well-known marine gastropod fauna from Europe. Indeed, these findings already highlighted several cases of pervasive ambiguity in species identities right here in our “marine backyard”, including possible species complexes that would benefit from further studies (e.g. *Nassarius incrassatus, Patella candei* and *Littorina saxatilis*), and cases of species and subspecies that are in need of taxonomic revisions (e.g. *Patella ulyssiponensis*, *P. aspera*, and *Tricolia pullus azorica*). These revisions and clarifications can be done more effectively “with a little help” from DNA barcoding, particularly because of the facility of comparing results from multiple studies and diverse locations.

## Methods

### Sampling, preservation and identification of specimens

**Figure 3:**
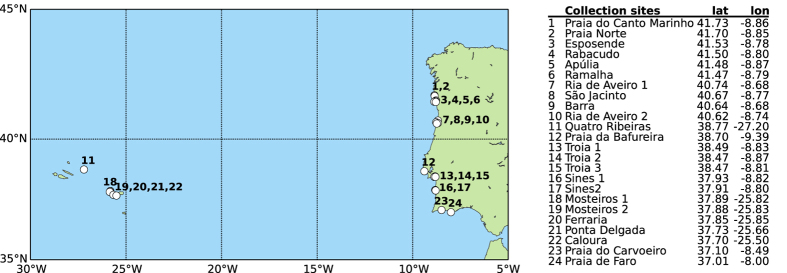
Location and coordinates (decimal degrees) of the 24 sites were gastropod specimens were collected from 2009 to 2015. The figure was created using python3/matplotlib.

Marine gastropod specimens were collected between 2009 and 2015, at sites along the coasts of mainland Portugal and two Azorean Islands, São Miguel and Terceira, Portugal (Fig. 3). Whenever possible, we collected 6 to 10 specimens per species, either on the intertidal by hand or on subtidal zones by SCUBA diving. After collection, specimens were preserved in 96–100% ethanol. To prevent dilution, the ethanol was changed 3 times after 24 hours of the initial preservation. In addition, each specimen was imaged and ancillary data were collected. These metadata are publicly available on the Barcode of Life Data system (BOLD)^19^, project Gastropoda of Portugal (GOP) in the campaign ‘Portugal Aquatic Life’. Specimens are stored at 4 °C in the Centre for Molecular and Environmental Biology, University of Minho for future reference. Voucher specimens will be deposited at the National Museum of Natural History (MNHN), in Lisbon.

All specimens collected were morphologically identified to genus or species level, initially, using the keys in Hayward & Ryland^35^ and in some cases consulting more specific works for the Portuguese fauna (e.g.^52^). The species’ nomenclature used in this work complies with the accepted nomenclature used in WoRMS and ITIS.

### DNA extraction, amplification and sequencing

Sequence data were generated in three institutions, Centre of Molecular and Environmental Biology (CBMA), University of Minho, Marine and Environmental Science Centre (MARE), Lisbon New University, Portugal, and the Biodiversity Institute of Ontario (BIO), Canada. At CBMA and MARE sequences were generated by extracting the total genomic DNA using GenElute Mammalian Genomic DNA Mini Prep kit (Sigma) or E.Z.N.A. Mollusc DNA Kit (Omega Bio-tek), following the protocol provided by the supplier. However, instead of the gene elute solution provided in the kit, ultrapure autoclaved water (50 μL) was used. We amplified a 658-bp fragment from the 5′ end of the mtDNA gene coding for cytochrome oxidase I (COI-5P) using the primer pairs LCO1490 and HCO2198^49^ and LoboF1 and LoboR1^27^. Amplifications were performed in 25 μL reactions, each reaction containing 2.5 μL of 10× Promega PCR buffer, 2.5 μL of MgCl_2_ (25 mM), 0.25 μL of dNTPs (10 mM), 0.5 μL of each primer (10 mM), 0.25 μL of Taq polymerase (5U/μL) and 2–6 μL of DNA template, filled up with ultrapure sterile H_2_O. Cycling conditions for PCR reactions with the Folmer primers were: one cycle of 94 °C for 1 min, five cycles of 94 °C for 30 s, 45 °C for 90 s and 72 °C for 60 s, 35 cycles of 94 °C for 30 s, 51 °C for 90 s and 72 °C for 60 s, with a final extension of 72 °C for 5 min. When using the Lobo primers the cycling conditions were: one cycle of 94 °C for 1 min, five cycles of 94 °C for 30 s, 45 °C for 90 s and 72 °C for 60 s, 45 cycles of 94 °C for 30 s, 54 °C for 90 s and 72 °C for 60 s, with a final extension of 72 °C for 5 min. Amplification success was screened in 1% agarose gel, using 5 μL of PCR product. Successful PCR products were then purified using a mix of 10U exonuclease I and 1U of shrimp alkaline phosphatase and subject to a thermal regime of 37 °C for 15 min and 80 °C for another 15 min. Cleaned-up amplicons were sent to an external sequencing service supplier (STAB Vida Ltd, Portugal), for bidirectional sequencing using the BigDye Terminator 3 kit, and run on an ABI 3730XL DNA analyser.

Tissue samples, obtained from the specimens provided by the Portuguese Institute of Malacology (IPM), were sent in a 96 well-plate to the Canadian Centre for DNA barcoding, Guelph, Canada, for COI-5 P amplification and sequencing. At BIO, the sequences were generated using the following procedure. A small piece of tissue was initially lysed in 45 μl cetyltrimethylammonium bromide (CTAB) and 5 μL proteinase K. The samples were then incubated at 56 °C for 12–18 h, followed by DNA extraction with a 3-μm glass fibre plate and re-suspended in 50 μL of ddH_2_0.The COI-5 P region was amplified using the primer set dgLCO-1490 and dgHCO-2198. PCR reactions were carried out in a total volume of 12.5 μL, containing 6.25 μL of 10% trehalose, 2 μL of ddH_2_0, 1.25 μL of 10 X PCR buffer, 0.625 μL of MgCl_2_ (50 mM), 0.125 μL of each primer (10 M), 0.0625 μL dNTPs (10 mM), 0.06 μL platinum Taq polymerase and 2 μL of DNA template (10–50 ng). The cycling conditions were: one cycle of 1 min at 94 °C, five cycles 40 s at 94 °C, 40 s at 45 °C, 1 min at 72 °C, 5 min at 72 °C, followed by 35 cycles of 40 s at 94 °C, 40 s at 51 °C and 1 min at 72 °C (Steinke, pers. com). PCR products were bi-directionally sequenced using BigDye v3.1 on an ABI 3730xl DNA Analyser.

### Data analysis

Complementary strands of COI sequences were edited and aligned manually using MEGA v. 6.1^57^. A multiple alignment was performed using Clustal W (also implemented in MEGA), checked for possible occurrence of indels (insertions and deletions), and subsequently translated into amino acid sequences to inspect for the presence of stop codons or unusual amino acid sequence patterns. In addition, we also used Muscle^58^ for multiple alignments to compare with the alignment produced using Clustal W. Sequence data and specimen metadata were uploaded in the project ‘Gastropoda of Portugal’ (GOP) within BOLD System, and submitted to GenBank (accessions numbers available in the project GOP publicly /available in the BOLD system). Following basic editing, the sequence similarity searches for species identification were carried out using BLASTN search at the National Centre for Biotechnology Information (NCBI), and BOLD Identification System tool (BOLD-IDS)^19^. The maximal levels of identity, expressed in percentages, of each submitted COI-5 P sequence in both public databases were compared. The Bayesian inference (BI) was conducted in MrBayes v.3.2^59^ to build the Bayesian tree with a total of 223 sequences (58 morpho-species), 109 generated in the present study (from 34 morpho-species), and 114 additional sequences (24 morpho-species) selected from GenBank. In order to compare and validate our results, we selected sequences from GenBank either from the same morpho-species collected in the same and in different locations (when existent) or from the same genera (supplementary Table S1). The BI topology was constructed choosing GTR + G + I as best-fitting model of nucleotide substitution based on its Bayesian Information Criterion as implemented in MEGA v.6.1. Posterior probabilities with >0.95 were considered robust. In addition, the neighbor-joining (NJ) method was used for building a phenogram using the K2P model, and the bootstrap support for the nodes was determined using 1000 iterations.

The BIN algorithm was applied by the BOLD system to produce Molecular Operational Taxonomic Units (MOTUs) that closely correspond to species^29^ and are represented by a unique number in the BOLD system (used in the present study). BINs were developed as an interim system to deal with the many species that await a formal description, and with other taxa that actually representing species complexes^10^. However, this automated system requires input from users and experts to validate and annotate the data associated with each MOTU^29^. Thus, all BINs were reviewed in the present work. Intra- and inter-specific divergence and nearest neighbour distance were calculated with the K2P model, the standard genetic distance used in DNA barcode studies, allowing direct comparisons with other DNA barcoding studies. In addition, species delimitation analyses were carried out using the Poisson-Tree Processes (PTP) approach. This model uses branch lengths to estimate the mean expected number of substitutions per site between two branching events^60^. The molecular species delimitations were conducted on the Web server of the Exelixis Lab (http://species.h-its.org/ptp/), using a model based gene tree generated with MrBayes and default settings. To provide a species-by-species measure of the taxonomic reliability of our DNA barcode records, we applied the ranking system proposed by Costa and co-authors^17^ and adapted to the use of BINs as MOTU delimitation criteria (e.g.^28^) (Fig. 2). The ranking varies from A to E, where “A” represents highly reliable species barcodes and “E” barcodes with lower reliability:

- **Grade A**: external concordance; unambiguous BIN match with all specimens of the same morpho-species from other BOLD projects or published sequences.
- **Grade B**: internal concordance; species in our dataset clustered in a single BIN, with at least 3 specimens of the same species examined. No matching sequences found from other studies.
- **Grade C**: sub-optimal concordance (possible genetic structure within species); at least 3 specimens of the same morpho-species are available within the library but they are divided in more than one BIN.
- **Grade D**: insufficient data; low number of specimens analysed (1 or 2 individuals) and no matching sequence available in BOLD or other public repositories.
- **Grade E**: discordant species assignments; sequences for a given species in our dataset did not match with the BIN or BINs for the same species in BOLD. The specimen may match with a BIN of a different species or be placed in a separate non-neighboring BIN.

## Additional Information

**How to cite this article**: Borges, L. M. S. *et al.* With a little help from DNA barcoding: investigating the diversity of Gastropoda from the Portuguese coast. *Sci. Rep.* **6**, 20226; doi: 10.1038/srep20226 (2016).

## Supplementary Material

Supplementary Information

## Figures and Tables

**Table 1:** Intra and interspecific K2P distances of gastropods species, genus and families analysed in this study.

Comparison (Intra-)	Taxa	Comparisons (N)	Minimum distance (%)	Mean distance (%)	Maximum distance (%)	SE (%)
Species	43	493	0	0.95	6.98	0
	**39**	**458**	**0**	**0.8**	**6.38**	**0**
Genus	11	1647	0	12.97	31.74	0
	**11**	**1163**	**2.51**	**13.32**	**31.74**	**0**
Family	5	1333	0	16.92	74.67	0.01
	**5**	**908**	**8.44**	**17.84**	**74.67**	**0.01**

Distance summary determined excluding cases of ambiguity are shown in bold.
