# Supplementary material for: With a little help from DNA barcoding: investigating the diversity of Gastropoda from the Portuguese coast
Source: Sci Rep. 2016 Feb 15;6:20226. doi: 10.1038/srep20226 (PMC4753432; doi:10.1038/srep20226)
Supplement: Supplementary Information [file srep20226-s1.pdf]

# With a little help from DNA barcoding: investigating the diversity of Gastropoda from the Portuguese coast

Luísa M. S. Borges, Claudia Hollatz, Jorge Lobo, Ana M. Cunha, Ana P. Vilela, Gonçalo Calado, Rita Coelho, Ana C. Costa, Maria S. G. Ferreira, Maria H. Costa, Filipe O. Costa

| Species name (no. of specimens) | GenBank accession no. | BOLD ID no. | source |
|---|---|---|---|
| *Acmae mitra* (1) | AB238459 | | Nakano & Ozawa, 2007 |
| *Armina maculata* (3) | KF369111 | **LMBRR022; LMBRR023** | This study; Lobo *et al*., 2013 |
| *Berthella plumula* (3) | KF369114;KF369115 | **LMBSCt6-002** | This study; Lobo *et al*., 2013 |
| Berghia columbina (1) | JX087544 | | Carmona *et al*., 2012 |
| *Berghia verrucicornis* (6) | HQ616750; JX087553; JX087554 | **LMBAG34-001; LMBAG34-002; LMBAG34-003** | This study; Carmona *et al*., 2011; Carmona *et al*., 2012 |
| Calliostoma sp. (1) | | LMBAG35-001 | This study |
| *Calliostoma virescens* (2) | | LMBAG35-002; LMBAG35-003 | This study |
| *Calliostoma zizyphinum* (6) | KR084610; KR054821; KR084401 | **TR1-15; TR2-14; TR2-16** | This study; Barco *et al*., 2015 |
| *Cingula trifasciata* (2) | | LMBAG12-002; LMBAG12-003 | This study |
| *Crepidula fornicata* (3) | AF353131; HM884254 | **AVP104** | This study; Colin, 2001 |
| *Euspira nitida* (2) | EU332641 | **TR1-26** | This study; Huelsken *et al*., 2008 |
| *Gibbula cineraria* (6) | AM049339; KJ183016 | **LMBAG22-001; LMBAG22-002; LMBAG22-004; LMBAG22-005** | This study; Williams & Ozawa, 2006; Cowart *et al.*, 2014 |
| *Gibbula delgadensis* (3) | | LMBAG9-001; LMBAG9-002; LMBAG9-003 | This study |
| *Gibbula sp.* (4) | | LMBV4-001; LMBV4-002; LMBV4-004; LMBV4-005 | This study |
| *Gibbula pennanti* (1) | GQ232365 | | Williams *et al*., 2010 |
| *Gibbula umbilicalis* (10) | JN686278; JN6863765; JN686273,GQ232367 | **LMBAG36-001; LMBAG36-002; LMBAP3-001; LMBAP3-002; AVP029; AVP069** | This study; Donald *et al*., 2012; Williams *et al*., 2010 |

| Species name (no. of specimens) | GenBank accession no. | BOLD ID no. | source |
|---|---|---|---|
| Gibbula umbilicaris (1) | JQ839383 | | Barco et al., 2013a |
| Gibbula varia (4) | JQ839392; JQ839395; JX887429; JX887427 | | Barco *et al*., 2013a |
| *Hexaplex trunculus* (5) | AM712610; AM712613; KF297431; EU391577 | **LMBAG28-001** | This study; Claremont *et al.*, 2008; Gonzalez-Tizon *et al*., 2011; Vilamor *et al*., 2014 |
| *Jujubinus exasperatus (1)* | GQ232368 | | Williams *et al*., 2010 |
| *Jujubinus pseudogravinae* (1) | | LMBAG14-001 | This study |
| *Littorina arcana (1)* | HE590832 | | Reid, Dyal & Williams, 2012 |
| *Littorina compressa (1)* | HE590834 | | Reid, Dyal & Williams, 2012 |
| *Littorina fabalis (1)* | HE590835 | | Reid, Dyal & Williams, 2012 |
| *Littorina littorea* (3) | EU876085 | **LMBAG7-002; LMBAG7-003** | This study; Blakeslee *et al*., 2008 |
| *Littorina obtusata* (4) | KF643904; AJ622947 | **LMBV18-003; LMBV18-005** | This study; Layton *et al*., 2014; Williams & Reid, 2004 |
| *Littorina saxatilis* (5) | KF643665; JN241971 | **LMBV17-002; LMBV17-003; LMBV18-004** | This study; Layton *et al*., 2014; Prado-Sanchez *et al*., 2011 |
| Lottia fascicularis (1) | AF130120 | | Simison & Lindberg, unpublished |
| *Lottia gigantea* (1) | AB238466 | | Nakano & Ozawa, 2007 |
| *Lottia strigatella (1)* | AF295539 | | Simison & Lindberg, unpublished |
| *Mitra cornea* (2) | | LMBSM2-002; LMBSM2-002 | This study |
| *Nassarius incrassatus* (7) | NEOGA1001; NEOGA1002; NEOGA1003 (BOLD system) | **LMBSM3-002; LMBSM3-003; LMBSM3-004; LMBV2-009** | This study; Puillandre & Gofas, unpublished |
| *Nassarius reticulatus* (7) | KJ183015; KF369156; KF369157 ;EU827201 | **LMBV2-004; LMBV10-001; LMBAG20-001** | This study; Cowart *et a*l, 2014; Lobo *et al*., 2013; Cunha *et al*., 2008 |
| *Niveotectura pallida* (1) | AB238494 | | Nakano & Ozawa, 2007 |
| *Nucella lapillus* (9) | DQ501718; FN651945; EU391582; KF644222 | **LMBAG19-001; LMBV7-001; LMBV7-002; LMBV7-003; LMBAP4-001** | This study; Guerra-Varela *et al*., 2007; Barco *et al.*, 2009; Claremont *et al*., 2008; Layton *et al*., 2014 |
| *Ocenebra erinaceus* (6) | KF369161; KF369162; KF369163 | **LMBV1-001 LMBV1-003 LMBV1-004** | This study; Lobo *et al*., 2013 |
| Ocinebrina aciculata (6) | KF199911; KF199917; KF199916; FR851900; FR851905; FR851906 | | Barco *et al*., 2013 |

| Species name (no. of specimens) | GenBank accession no. | BOLD ID no. | source |
|---|---|---|---|
| *Ocinebrina edwardsii* (6) | KF153478; KF153443; KF153303; KF153263 | **LMBAG23-001; LMBAG23-002** | This study; Barco *et al*., 2013 |
| *Ocinebrina hispidula* (3) | KC883664; KF153274; KF153302 | | Barco *et al*., 2013 |
| *Ocinebrina ingloria* (3) | KF153316; KF153394; KF153437 | | Barco *et al*., 2013 |
| *Ocinebrina leukos* (1) | KF153286 | | Barco *et al*., 2013 |
| *Ocinebrina lurida* (1) | KF643785 | | Layton *et al*., 2014 |
| *Ocinebria miscowichae* (3) | KF153346; KF153350; KF153417 | | Barco *et al*., 2013 |
| *Ocinebrina nicolai* (3) | KF153446; KF153281; KF153434 | | Barco *et al*., 2013 |
| *Ocinebrina piantonii* (1) | KF153271 | | Barco *et al*., 2013 |
| Ocinebrina sp. (1) | | LMBS12 | This study |
| *Patella aspera* (6) | EF462971; EF462966 | **LMBT5-001 LMBT5-002 LMBT5-003; LMBSM5-001** | This study; Borrell *et al*., 2007 |
| *Patella candei* (9) | DQ089564; DQ089570; DQ089573 | **LMBT1-001; LMBT1-003; LMBT1-004; LMBSM8-001; LMBSM8-002; LMBSM8-003** | This study; Sá-Pinto *et al*., 2008; Sá-Pinto *et al*., 2005 |
| *Patella depressa* (9) | JF937119; JF937155 | **LMBAG33-001; LMBAG33-002; LMBAG25-001; LMBAG25-002; LMBAG25-003; LMBAP1-001, LMBAP1-002** | This study; Munoz-Comenero et al., 2011 |
| Patella ferruginea (1) | AB238578 | | Nakano & Ozawa, 2007 |
| *Patella pellucida* (2) | DQ089620 | **LMBV15-001** | This study; Sá-Pinto *et al*., 2005 |
| *Patella rustica (1)* | AB238579 | | Nakano & Ozawa, 2007 |
| *Patella ulyssiponensis* (7) | HF547539; HF547517; HF547528; DQ089596 | **LMBV9-001, LMBV9-002; LMBV9-003** | This study; Sá-Pinto *et al.*, 2008; Sá-Pinto *et al*., 2005 |
| *Patella vulgata* (8) | JF937168; AB238589 | **LMBV22-001; LMBV22-002; LMBV23-001; LMBV23-002; LMBV23-003; LMBV24-001** | This study; Munoz-Comenero *et al*., 2011; Nakano & Ozawa, 2007 |
| *Phorcus lineatus* (13) 100 | JN686331; JN686317; JN686329 | **LMBAG4-003; LMBAG21-001; LMBAG21-002; LMBAG21-003; LMBAG32-001; LMBAG32-002; LMBAG32-003; LMBAG38-002; LMBA1-002; LMBV4-003** | This study; Donald *et al*., 2012 |
| *Phorcus sauciatus* (4) | JN686300; JN686303;KF369166; KC990630 | | Donald *et al*., 2012; Lobo *et al*., 2013; Galindo *et al*., 2014 |
| *Scurria bahamondina (*1) | AB238531 | | Nakano & Ozawa, 2007 |
| *Siphonaria pectinata (9)* | HQ386635; HQ386652; HQ3886654; HQ386639 | **LMBAG18-001; LMBAG18-002; LMBAG18-003; LMBAP2-001; LMBAP2-002** | This study; Kawauchi & Giribet, 2011 |

| **Species name (no. of specimens)** | **GenBank accession no.** | **BOLD ID no.** | **source** |
|---|---|---|---|
| *Tectarius striatus* (3) | KC990635 | **LMBT2-001; LMBSM6-006** | This study; Galindo *et al*., 2014 |
| Tectura virginea (2) | AB238541 | **LMBV8-001** | This study; Nakano & Ozawa, 2007 |
| *Tricolia pullus* (1) | AM049358 | | Williams & Ozawa, 2006 |
| *Tricolia pullus azorica* (2) | | LMBAG10-001; LMBAG10-002 | This study |
| *Vermetus triquetrus* (1) | KF369193 | | Lobo *et al*., 2013 |
